# Tuberculous Meningitis in a One-Year-Old Child With Isoniazid-Induced Hepatotoxicity: A Case Report

**DOI:** 10.7759/cureus.89383

**Published:** 2025-08-04

**Authors:** Satoshi Okada, Shinsuke Mizuno, Nobuyuki Akutsu, Hiroshi Kurosawa, Masashi Kasai

**Affiliations:** 1 Division of Pediatric Critical Care Medicine, Hyogo Prefectural Kobe Children's Hospital, Hyogo, JPN; 2 Division of Infectious Diseases, Hyogo Prefectural Kobe Children’s Hospital, Hyogo, JPN; 3 Division of Neurosurgery, Hyogo Prefectural Kobe Children’s Hospital, Hyogo, JPN; 4 Division of Pediatric Critical Care Medicine, Hyogo Prefectural Kobe Children’s Hospital, Hyogo, JPN; 5 Division of Infectious Diseases, Hyogo Prefectural Kobe Children's Hospital, Hyogo, JPN

**Keywords:** children, hepatotoxicity, isoniazid, levofloxacin, linezolid, tuberculous meningitis

## Abstract

Tuberculous meningitis (TBM) is predominantly observed in developing countries but remains relatively rare in developed countries. Therefore, if a clinician does not suspect TBM, its diagnosis may be delayed. Furthermore, drug-induced hepatotoxicity is common and can become severe during TBM treatment. Given the importance of multidrug regimens for TBM management, alternative drugs with favorable cerebrospinal fluid (CSF) penetration and high safety in terms of side effects are urgently required. We report a case of a one-year and 10-month-old Japanese boy who presented with an eight-day history of fever and altered consciousness. Contrast-enhanced magnetic resonance imaging revealed brainstem infarction, hydrocephalus, and basilar meningeal enhancement. CSF analysis showed an increased cell count with a predominance of mononuclear cells. On the basis of these findings, we suspected TBM and initiated antituberculosis treatment, including isoniazid, rifampicin, ethambutol, and pyrazinamide, along with steroids and aspirin. TBM was confirmed based on a combination of clinical findings and a positive sputum culture for *Mycobacterium tuberculosis*. During treatment, the patient developed isoniazid-induced hepatotoxicity, characterized by elevated levels of hepatic transaminases and hyperbilirubinemia. Substituting isoniazid with linezolid and levofloxacin in the initial treatment successfully ameliorated the hepatic injury without additional adverse events.

This suggests that even in developed countries, clinicians must maintain a high suspicion of TBM when evaluating children with subacute neurological symptoms and consider performing additional imaging studies and CSF examinations. Further, this case demonstrated that linezolid and levofloxacin can be useful alternatives to isoniazid in preventing associated hepatotoxicity.

## Introduction

Tuberculosis (TB) remains a significant epidemiological threat to children worldwide. Approximately 1.25 million children under the age of 15 years are infected with TB annually, with approximately 210,000 (16%) deaths [1]. It is particularly concerning that children under the age of five years account for approximately 40% of all pediatric TB cases and 76% of TB-related deaths among HIV-negative children [1].

Tuberculous meningitis (TBM), characterized by symptoms such as low-grade fever, malaise, headache, and hydrocephalus, is commonly observed in developing countries, accounting for approximately 10% of pediatric TB cases [2]. However, it is relatively rare in developed countries, accounting for less than 3% of estimated annual bacterial meningitis cases, with 100-150 cases reported annually in the United States [3]. Similarly, Japan reports only zero to three TBM cases annually [4]. Delays in the diagnosis and treatment of TBM negatively affect prognosis and could result in neurologic sequelae and mortality, emphasizing the importance of early detection and intervention [3]. TB is treated with a combination of several anti-TB drugs, including isoniazid, the major adverse effect of which can be hepatotoxicity. Moreover, patients with TBM have been reported to be more susceptible to liver damage compared with patients having pulmonary TB [5].

Here, we report a case of a young child who developed TBM and presented with an eight-day history of fever and altered consciousness; furthermore, imaging and cerebrospinal fluid (CSF) tests showed typical findings. During treatment, the patient developed isoniazid-induced hepatotoxicity, necessitating initial treatment with linezolid and levofloxacin. To better contextualize the patient’s clinical course and treatment, we conducted a narrative review of published literature on TBM in children, with a particular emphasis on alternative regimens involving linezolid and levofloxacin, assessing their safety, efficacy, and CSF penetration.

## Case presentation

A Japanese boy aged one year and 10 months presented to a local clinic with a two-day history of fever and lethargy. Because he did not present with respiratory symptoms, and influenza and SARS-CoV-2 test results were negative. The patient was initially diagnosed with the common cold. His fever reduced in two days, but lethargy persisted, and he developed involuntary movements. Therefore, he was brought to a local general hospital eight days after symptom onset. Brain magnetic resonance imaging (MRI) revealed hydrocephalus, leading to the suspicion of a brain tumor, and the patient was transferred to our hospital.

The patient had no relevant medical history and showed normal development. The family was entirely Japanese, with no recent history of traveling abroad, including TB-endemic countries, and the child was the fourth of five siblings. Six months earlier, his father had a fever for two weeks, followed by a persistent cough. The siblings also had frequent fevers and coughs in the past few months. The patient had been administered the Bacillus Calmette-Guérin vaccine at six months of age without a history of Koch’s phenomenon.

The patient’s vital signs on admission were as follows: respiratory rate, 30 breaths/minute; oxygen saturation, 99% (room air); heart rate, 107 beats/minute; systolic blood pressure, 120 mmHg; and body temperature, 37.9℃. The pediatric Glasgow Coma Scale score was E2V3M4. Physical examination revealed nuchal rigidity, positive Brudzinski sign, and increased tendon reflexes and muscle tone. Laboratory findings at admission showed a slight increase in white blood cell count and C-reactive protein value (Table 1).

**Table 1:** Laboratory findings and cerebrospinal fluid analysis on admission ALT: alanine aminotransferase; AST: aspartate aminotransferase; Cl: chloride; Cr: creatinine; CRP: C-reactive protein; Hb: hemoglobin; K: potassium; Na: sodium; PLT: platelet count; T-bil: total bilirubin; UN: urea nitrogen; WBC: white blood cell count

Test	Result	Unit	Reference range
Hematology			
WBC	1.47 × 10^9^	/L	4.3-19.6
Hb	107	g/L	105-141
PLT	71.4 × 10^9^	/L	16.8-65.0
Serology			
CRP	2.1	mg/L	0-3
Biochemistry			
AST	18	U/L	23-57
ALT	8	U/L	9-38
T-bil	5.13	μmol/L	3.42-12.0
UN	3.75	mmol/L	2.86-7.14
Cr	18.6	μmol/L	12.4-30.9
Na	132	mmol/L	135-143
K	4.9	mmol/L	3.6-5.1
Cl	93	mmol/L	101-110
Glucose	6.55	mmol/L	3.33-6.11
Cerebrospinal fluid findings			
Cell counts	72 × 10^6^	/L	0-8
Monoclear	10	%	-
Glucose	2.39	mmol/L	1.89-6.61
Protein	950	g/L	200-1,700
Adenosine deaminase	4.4	U/L	0-4

**Figure 1:**
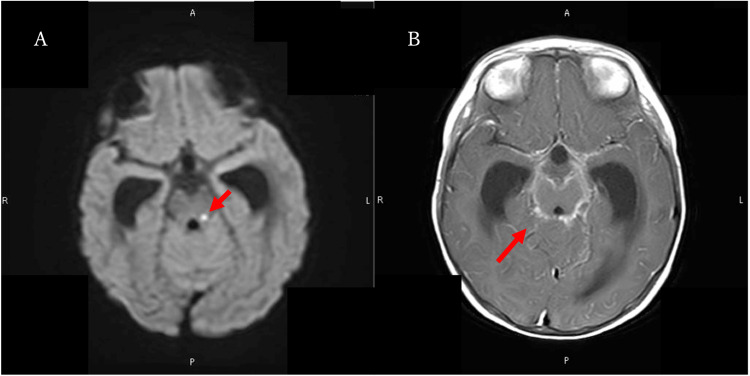
Brain magnetic resonance imaging performed at admission. (A) Diffusion-weighted imaging demonstrating infarction in the midbrain (arrow). (B) Contrast-enhanced T1-weighted magnetic resonance imaging showing enhancement along the brainstem in the basilar cisterns and sulci (arrow)

Contrast-enhanced MRI of the brain showed infarcts in the ventral thalamus and pons and bilateral ventricular and third ventricle expansion, with enhancement of the basal cisterns and sulci (Figure 1).

**Figure 2:**
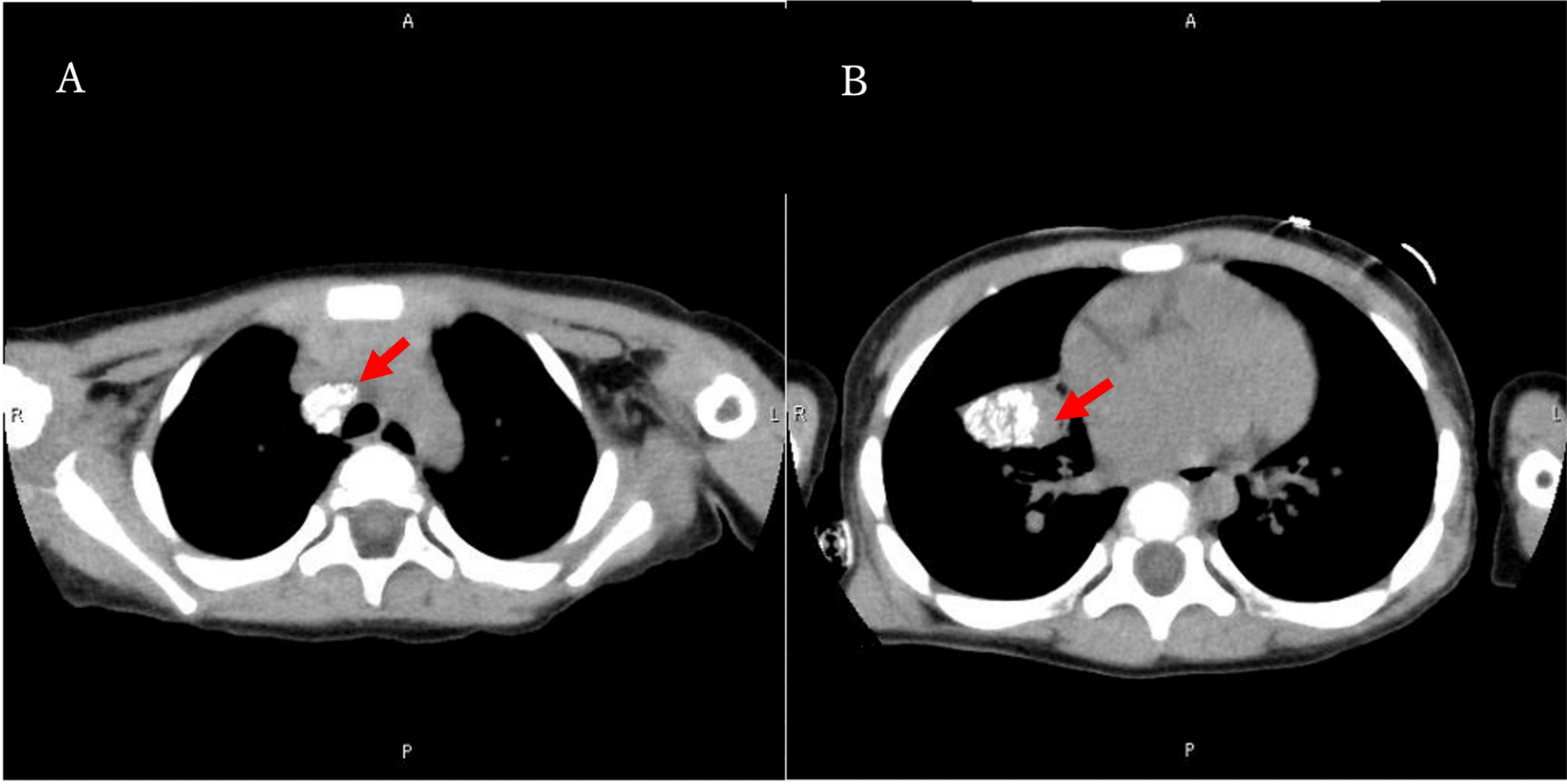
Chest computed tomography images obtained at admission. (A) Calcified lymph nodes were observed in the right pulmonary hilar region (arrow). (B) A calcified mass was detected in the right lung (arrow)

Chest radiography revealed a right pulmonary hilar mass, and chest computed tomography showed a calcified mass with calcified lymph nodes extending from the right pulmonary hilum to the mediastinum (Figure 2).

After admission, an external ventricular drain was placed to treat the hydrocephalus. CSF analysis showed an increased mononuclear cell count, an elevated protein level, and a decreased glucose level (Table 1). CSF smear and multiplex polymerase chain reaction tests were negative. Based on these findings, TBM was suspected, and treatment with steroids and aspirin was initiated for associated basilar arteritis. The patient’s sputum culture was positive for *Mycobacterium tuberculosis*, leading to a definitive diagnosis of TBM. Acid-fast bacillus smears, polymerase chain reaction, and culture tests of the CSF specimens were all negative. Drug susceptibility testing for *M. tuberculosis* showed sensitivity to all tested agents. The sputum culture of his father was also positive for *M. tuberculosis*.

**Figure 3:**
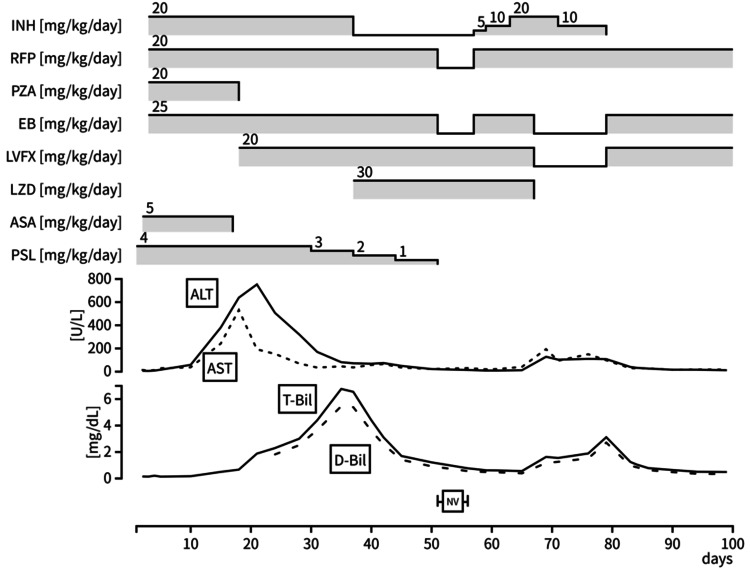
Patient’s clinical course during hospitalization From days 51 to 55, owing to the patient’s gastrointestinal symptoms, which were attributable to norovirus infection, only intravenously administered levofloxacin and linezolid were maintained ALT: alanine aminotransferase; ASA: aspirin; AST: aspartate aminotransferase; D-Bil: direct bilirubin; EB: ethambutol; INH: isoniazid; LVFX: levofloxacin; LZD: linezolid; NV: norovirus; PSL: prednisolone; PZA: pyrazinamide; RFP: rifampicin; T-Bil: total bilirubin

Anti-TB treatment was initiated on day 2 by using a four-drug regimen: isoniazid 20 mg/kg/day, rifampicin 20 mg/kg/day, ethambutol 25 mg/kg/day, and pyrazinamide 40 mg/kg/day. Following treatment initiation, the CSF cell count reduced, but aspartate aminotransferase and alanine aminotransferase levels were elevated to more than five times the upper limit of normal, reaching approximately 300 U/L. Abdominal ultrasound revealed no abnormalities, and drug-induced hepatotoxicity was suspected; aspirin was discontinued, and pyrazinamide was replaced with levofloxacin (20 mg/kg/day). Hepatic transaminase levels improved; however, total bilirubin levels increased. On day 34, the total bilirubin level reached 6.8 mg/dL (normal range: 3.42-12.0 mg/dL), with the direct bilirubin level being 5.5 mg/dL (normal range: 0.00-0.40 mg/dL) (Figure 3).

Considering the hepatotoxic effects of isoniazid, the drug was replaced with linezolid (30 mg/kg/day). The patient’s hyperbilirubinemia was subsequently alleviated, and the initial treatment with rifampicin, ethambutol, levofloxacin, and linezolid was continued.

On day 57, isoniazid was reintroduced at a dose of 5 mg/kg/day, and its dose was gradually increased. After confirming the absence of hepatic complications at the original dosage of 20 mg/kg/day, two-drug maintenance therapy with isoniazid and rifampicin was initiated on day 67. However, the aspartate aminotransferase, alanine aminotransferase, and bilirubin levels increased again, suggesting isoniazid-induced hepatotoxicity. Although the dosage of isoniazid was reduced to 10 mg/kg/day, bilirubin levels continued to increase. On day 79, the regimen was changed to ethambutol, rifampicin, and levofloxacin, which resolved the hyperbilirubinemia. Maintenance therapy was adjusted to three drugs (ethambutol, rifampicin, and levofloxacin) for four months, followed by two drugs (ethambutol and rifampicin) for six months.

Basilar enhancement on MRI resolved on day 22. For hydrocephalus management, an endoscopic third ventriculostomy was performed on day 36. Owing to increased vomiting, a ventriculoperitoneal shunt was placed on day 66. Shunt revision was required on day 86 owing to shunt obstruction.

Neurological evaluation on day 113 (at the age of two years and two months) revealed significant developmental regression to the three-month developmental level. The child struggled with sitting by himself, exhibited right-sided limb rigidity, and had difficulty grasping with the right hand.

Follow-up treatment and rehabilitation were continued at a local general hospital. At 10 months after onset, the patient had no further hepatic injury, and his CSF cultures remained negative. While right upper limb rigidity and right lower limb paralysis persisted, by the time the patient reached the age of two years and eight months, the developmental delay improved to a level corresponding to the age of one year and two months. The father and one of the older siblings were treated for pulmonary TB, and two of the siblings were treated for tuberculous lymphadenitis.

## Discussion

We encountered a case of TBM in a young child with an eight-day history of fever and altered consciousness; the patient presented with typical findings of TBM on contrast-enhanced MRI and CSF examination. Although *M. tuberculosis* was not detected in the CSF culture, the diagnosis of TBM was made based on the presence of CSF pleocytosis with mononuclear cell predominance (≥10/mm³), elevated CSF protein levels (≥50 mg/dL), a CSF-to-serum glucose ratio of less than 0.5, and a positive sputum culture for *M. tuberculosis*. He developed isoniazid-induced hepatotoxicity during treatment and successfully completed the initial therapy with linezolid and levofloxacin.

Delayed treatment of TBM can lead to neurological sequelae and mortality [3]. However, in developed countries such as Japan, diagnosis and treatment initiation may be delayed owing to the low incidence of pediatric TBM. As people from highly endemic countries enter developed countries [6], the number of children diagnosed with TBM may increase. Therefore, pediatricians should be thoroughly familiar with the clinical manifestations of TBM.

The clinical course of TBM is typically subacute, with a median symptom duration of 10 days (range, one day to nine months) before diagnosis [3]. The predominant symptoms are low-grade fever, malaise, headache, and hydrocephalus. Nuchal rigidity may be absent, particularly in younger patients [3]. These nonspecific symptoms make early diagnosis challenging. Thus, imaging studies, particularly contrast-enhanced brain MRI, are useful for diagnosing and assessing complications [7]. The MRI findings of TBM include basilar meningeal enhancement and hydrocephalus with potential brainstem infarctions [7]. For patients presenting with prolonged neurological findings for a few weeks, clinicians should 1) investigate the travel history to TB-endemic regions, 2) investigate if family members have had prolonged fever and cough, 3) check Bacillus Calmette-Guérin vaccination status, and 4) consider imaging studies. When basilar meningeal enhancement or brainstem infarction is observed on MRI, CSF examination, which may also give characteristic findings of TBM, and early anti-TB treatment are recommended.

During anti-TB treatment, drug-induced hepatotoxicity is a frequent and severe adverse reaction. While less common in children compared with adults, pediatric studies report drug-induced liver injury in 7%-27% of cases of TB treatment with isoniazid, rifampicin, and pyrazinamide [8]. Drug-induced hepatotoxicity can lead to poor outcomes, necessitating prompt intervention when identified. TBM is the most severe form of the disease, and specific management strategies are recommended for hepatic injury [9]. These include 1) initial discontinuation of pyrazinamide, 2) discontinuation of isoniazid and rifampicin when hepatic dysfunction persists, and 3) use of fluoroquinolone antibiotics as an alternative.

In the case of our patient, isoniazid discontinuation improved the liver function test results. He showed signs of liver dysfunction again upon reinitiation of isoniazid. Therefore, isoniazid was considered responsible for liver injury. Although isoniazid is a key anti-TB drug, it can induce hepatotoxicity, particularly in pediatric patients with TBM. The frequency of isoniazid-induced hepatic injury varies significantly among populations. In pediatric populations receiving preventive treatment or non-TBM therapy, rates of hepatotoxicity are generally low (jaundice: 0.06%-0.83% and liver enzyme elevation: 8%) [5]. However, in TBM cases, these rates are substantially higher (jaundice, 10%; liver enzyme elevation, 52.9%) [5].

The onset of isoniazid-induced hepatotoxicity can occur as early as one week and as late as one year after treatment initiation [10]. Although symptoms typically improve within one week of drug discontinuation, in approximately 10% of cases, the condition can progress to severe hepatic injury [10].

Although the precise mechanism of hepatotoxicity remains unclear, toxic metabolites are believed to play an important role [11]. In the liver, N-acetyltransferase 2 acetylates isoniazid to acetylisoniazid, generating metabolites such as acetylisoniazid, hydrazine, and acetylhydrazine. Oxidative free radicals from these metabolites interact with hepatic macromolecules, causing toxicity and resulting in liver cell death [11]. The NAT metabolic pathway is genetically categorized into the rapid and slow acetylation groups. Compared with rapid acetylators, slow acetylators are more prone to hepatic injury owing to the increased production of toxic metabolites, such as hydrazine [11]. Dose adjustments did not improve hepatic injury in the present case, and delayed genetic acetylation may have contributed to the hepatotoxicity. However, there was no evidence for this case because genetic testing was not done.

Linezolid and levofloxacin are considered second-line drugs for drug-resistant TBM because of their favorable CSF penetration. Li et al. conducted a retrospective study of patients under 15 years of age with TBM and found that groups treated with standard regimens of linezolid demonstrated higher treatment success rates and shorter time to defervescence, with no significant difference in adverse events compared with the control group [12].

Regarding levofloxacin, although evidence for its use in infant TBM remains limited, a systematic review of 1,115 patients (including patients older than 15 years of age) found no improvement in mortality after fluoroquinolones were added to standard regimens or ethambutol and rifampicin were replaced with fluoroquinolones [13]. Heemskerk et al. reported the potential benefits of enhanced treatment with high-dose rifampicin and levofloxacin, specifically in patients with isoniazid-resistant TBM [14]. While some previous studies and reviews have suggested that quinolone antibiotics are safe for pediatric patients, there are concerns about potential cartilage damage from these drugs, resulting in their use in pediatric patients being discouraged [15,16]. However, levofloxacin may be an effective alternative if isoniazid cannot be administered. The patient’s family was thoroughly counseled about potential joint complications, and informed consent was obtained. In the present case, no joint-related adverse events were observed during levofloxacin administration.

Although the patient achieved microbiological resolution, he experienced profound developmental regression and persistent neurological deficits, including limb rigidity and delayed motor skills. These sequelae are consistent with reported complications of TBM when diagnosis and treatment are delayed, particularly in cases presenting with hydrocephalus and infarction. Previous studies report that up to 50% of pediatric TBM survivors exhibit long-term neurodevelopmental impairments [3,9]. This underscores the necessity for rapid recognition, early imaging, and aggressive multidisciplinary management, including neurosurgical and rehabilitative support, to minimize lasting neurological damage.

## Conclusions

We experienced a case of infant with subacute altered consciousness, typical CSF and MRI findings of TBM. Because treatment and diagnostic delays of TBM can worsen neurological outcomes, even in countries with low endemicity, pediatricians should consider TBM when children present with subacute neurological symptoms and should perform contrast MRI and CSF examinations. Furthermore, in pediatric TBM with isoniazid-induced hepatotoxicity, linezolid and levofloxacin can serve as initial treatment alternatives. Further studies are needed to understand the effectiveness and long-term safety of these drugs in pediatric patients with TBM.

## References

[1] (2024). Global tuberculosis report. https://iris.who.int/bitstream/handle/10665/373828/9789240083851-eng.pdf?sequence=1.

[2] Abdella A, Deginet E, Weldegebreal F, Ketema I, Eshetu B, Desalew A (2022). Tuberculous meningitis in children: treatment outcomes at discharge and its associated factors in Eastern Ethiopia: a five years retrospective study. Infect Drug Resist.

[3] Marx GE, Chan ED (2011). Tuberculous meningitis: diagnosis and treatment overview. Tuberc Res Treat.

[4] (2024). Ministry of Health, Labour and Welfare: Tuberculosis Registrar Information Survey Annual Report tabulation results in 2023. https://www.mhlw.go.jp/content/10900000/001295037.pdf.

[5] Donald PR (2011). Antituberculosis drug-induced hepatotoxicity in children. Pediatr Rep.

[6] Lo Vecchio A, Smarrazzo A, Amato C (2020). Increasing tuberculosis rates and association with migration in children living in Campania region, Southern Italy: a 10-year cohort study. Pediatr Infect Dis J.

[7] Oztoprak I, Gümüs C, Oztoprak B, Engin A (2007). Contrast medium-enhanced MRI findings and changes over time in stage I tuberculous meningitis. Clin Radiol.

[8] Gafar F, Arifin H, Jurnalis YD, Yani FF, Fitria N, Alffenaar JC, Wilffert B (2019). Antituberculosis drug-induced liver injury in children: incidence and risk factors during the two-month intensive phase of therapy. Pediatr Infect Dis J.

[9] Thwaites G, Fisher M, Hemingway C, Scott G, Solomon T, Innes J (2009). British Infection Society guidelines for the diagnosis and treatment of tuberculosis of the central nervous system in adults and children. J Infect.

[10] LiverTox LiverTox (2012). Clinical and Research Information on Drug-Induced Liver Injury. https://www.ncbi.nlm.nih.gov/books/NBK547852/.

[11] Sankar J, Chauhan A, Singh R, Mahajan D (2024). Isoniazid-historical development, metabolism associated toxicity and a perspective on its pharmacological improvement. Front Pharmacol.

[12] Li H, Lu J, Liu J, Zhao Y, Ni X, Zhao S (2016). Linezolid is associated with improved early outcomes of childhood tuberculous meningitis. Pediatr Infect Dis J.

[13] Rizvi I, Malhotra HS, Garg RK, Kumar N, Uniyal R, Pandey S (2018). Fluoroquinolones in the management of tuberculous meningitis: systematic review and meta-analysis. J Infect.

[14] Heemskerk AD, Bang ND, Mai NT (2016). Intensified antituberculosis therapy in adults with tuberculous meningitis. N Engl J Med.

[15] Chauny JV, Lorrot M, Prot-Labarthe S, De Lauzanne A, Doit C, Géréral T, Bourdon O (2012). Treatment of tuberculosis with levofloxacin or moxifloxacin: report of 6 pediatric cases. Pediatr Infect Dis J.

[16] Noel GJ, Bradley JS, Kauffman RE (2007). Comparative safety profile of levofloxacin in 2523 children with a focus on four specific musculoskeletal disorders. Pediatr Infect Dis J.

